# Global, regional, and national burden of children and adolescents with acute lymphoblastic leukemia from 1990 to 2021: a systematic analysis for the global burden of disease study 2021

**DOI:** 10.3389/fpubh.2025.1525751

**Published:** 2025-01-29

**Authors:** Lei Wang, Xue Yao, Linhua Yang

**Affiliations:** Department of Hematology, The Second Hospital of Shanxi Medical University, Taiyuan, China

**Keywords:** ALL, children and adolescents, deaths, DALYs, GBD 2021

## Abstract

**Background:**

Acute lymphoblastic leukemia (ALL) is a common cancer in children and adolescents, severely affecting their survival and health. With the discovery of new drugs and improved treatment options, the survival rate of ALL in children and adolescents has improved significantly.

**Methods:**

We used the GBD (global burden of disease) database to collect patients aged 0–19 years (0–5 years, 5–9 years, 10–14 years, 15–19 years) diagnosed with ALL between 1990 and 2021. Disease status and change were analyzed by learning about the prevalence, death, incidence, DALYs (disability-adjusted life years), percentage change, and the estimated annual percentage change (EAPC).

**Results:**

In 2021, there were 53,485 new cases of childhood and adolescent ALL, 23,991 deaths, and an estimated 1960,922 DALYs. Incidence, deaths and DALYs have declined globally, with only a rise in low-SDI regions. In 2021, middle-SDI regions have the highest cases of prevalence, incidence, deaths, and DALYs, accounting for approximately one-third of the global total. High-SDI regions have the lowest deaths and DALYs. East Asia has the highest prevalence and incidence. Australasia has the lowest death and DALYs. From 1990 to 2021, children and adolescent ALL deaths and DALYs show a declining trend in about 72.5% of countries, with only Sub-Saharan Africa showing an increase. The prevalence of ALL in children and adolescents is higher in males, with the highest cases in patients under 5 years of age.

**Conclusion:**

Our study highlights the trend of decreasing deaths and DALYs of ALL in children and adolescents. However, there is a need to improve healthcare prevention and timely standardized treatment in developing countries and less developed regions.

## Introduction

Acute lymphoblastic leukemia (ALL) is the most common and curable malignancy in children. There has been a small increase in the incidence of ALL in recent years, with approximately 60% of cases diagnosed in individuals before the age of 20 (1). Advances in drug discovery and the optimization of therapeutic regimens have significantly improved survival rates for children and adolescents with ALL. Over the past few decades, extensive clinical trials have been conducted, leading to notable enhancements in patient outcomes (2). In the last decade, the five-year survival rate for children aged 0–14 years has surpassed 90% in most high-income countries (3, 4). Conversely, survival rates remain low in low-and middle-income countries (5). Acute lymphoblastic leukemia continues to pose a significant threat to the survival of children and adolescents worldwide, underscoring the need for targeted efforts to enhance cure rates globally. Knowing the global incidence and survival rates of ALL in children and adolescents is important for improving medical care and treatment strategies.

This study employs the global burden of disease (GBD) 2021 database to analyze the prevalence, incidence, death, and disability-adjusted life years (DALYs) associated with childhood and adolescent ALL, along with their trends at global, regional, and national levels from 1990 to 2021. Stratified the data based on age, sex, country, and region, and by examining the impact of the Sociodemographic Index (SDI) at both regional and national levels, this research aims to establish a scientific foundation for the development of targeted interventions and relevant policies.

### Methods

We collected data on childhood and adolescent ALL from the global burden of disease database, which includes a wide range of information on global, regional, and national prevalence, incidence, deaths, and DALYs rates, percentage change, and the estimated annual percentage change (EAPC) from 1990 to 2021.

ALL in children and adolescents were divided into four age groups: <5 years, 5–9 years, 10–14 years, and 15–19 years. In addition, the 204 countries and 21 regions in the study were classified into five categories from low SDI to high SDI. Furthermore, the sociodemographic index (SDI), a measure of an area’s sociodemographic advancement based on income, education, and fertility, was employed in the study (6, 7).

To understand the trend of data over time, we used joinpoint regression analysis to construct the simplest model of the data by connecting multiple line segments (the so-called joinpoints) on a logarithmic scale. The analysis was conducted using Joinpoint software provided by the National Cancer Institute and Information Management Services. All procedures for analysis and graphic representation were performed utilizing the World Health Organization’s Health Equity Assessment Toolkit and the statistical computing software, R (Version 4.4.1), to generate internally consistent estimates, providing a 95% uncertainty interval (95% UI). And the ggplot2 package was used to create visualizations.

## Results

### Global level

**Figure 1:**
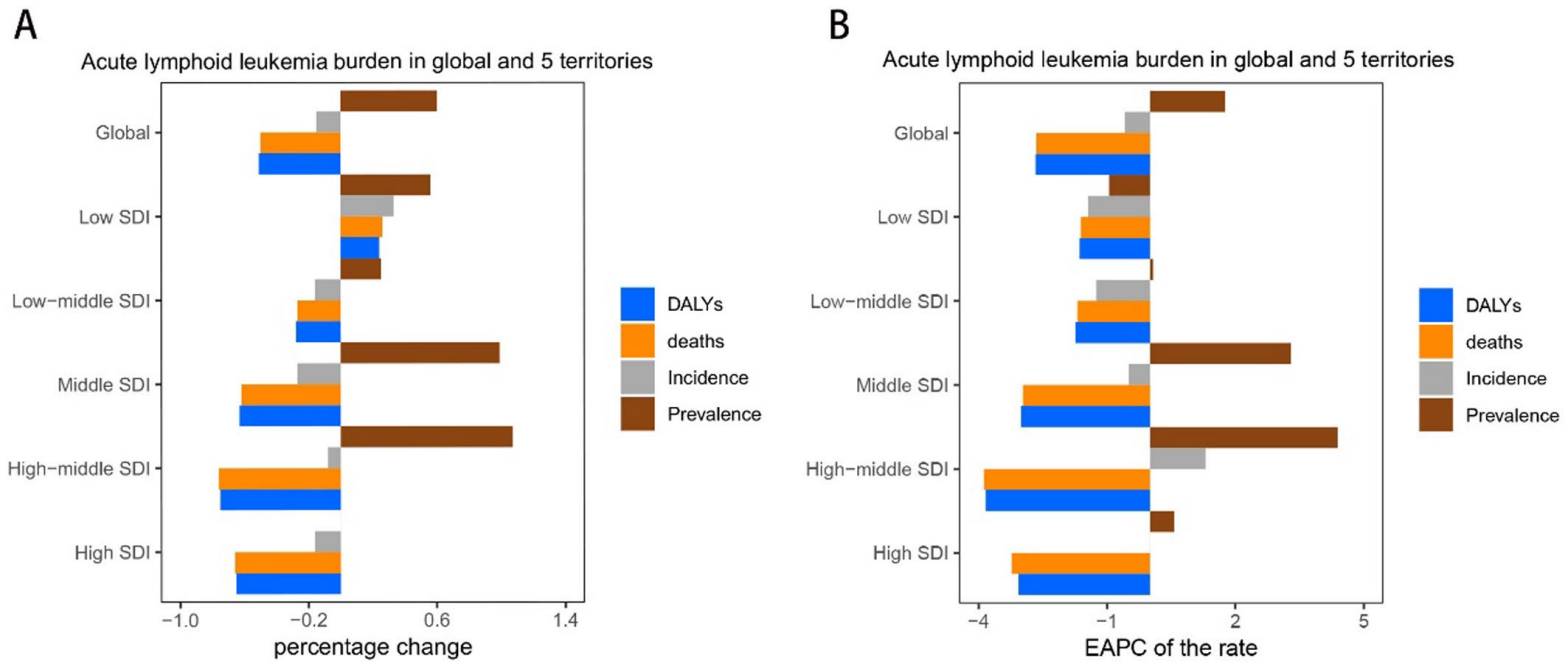
Trend of childhood and adolescent ALL burden in global and 5 territories from 1990 to 2021. **(A)** Percentage change in cases of prevalent, incidence, deaths, and DALYs in 1990 and 2021. **(B)** The EAPC of prevalence, incidence, deaths, and DALY rates from 1990 to 2021.

The global prevalence of ALL cases in children and adolescents has been reported to be on the rise, however, incidence, deaths, and DALYs have declined. The prevalence has increased from 178,730 cases in 1990 to 285,095 cases in 2021, a percentage change of 60%. Incidence decreased from 62,687 cases in 1990 to 53,485 cases in 2021. Deaths decreased from 48,061 cases in 1990 to 23,991 cases in 2021. DALYs decreased from 3,973,113 in 1990 to 1,660,921 in 2021. The change of the rates is consistent with the cases percentage change from 1990 to 2021 of the ALL in children and adolescents globally, with the EAPC of 1.76 (95% CI: 1.49–2.04), −0.59 (95% CI: −0.79 to 0.4), −2.65 (95% CI: −2.72 to 2.58), and −2.67 (95% CI: −2.75 to 2.59), respectively (Table 1; Figure 1; Supplementary Tables S1, S2).

**Table 1:** The prevalence of childhood and adolescent ALL cases and rates in 1990 and 2021, and the trends from 1990 to 2021.

	Location	Num_1990 (95% UI)	Num_2021 (95% UI)	Percentage change (100%)	Rate_1990 per 100,000 (95% UI)	Rate_2021 per 100,000 (95% UI)	EAPC_(95%CI)
1	Global	178730.12 (152374.73–217308.64)	285095.22 (192351.79–375929.59)	0.6	7.91 (6.75–9.62)	10.82 (7.3–14.26)	1.76 (1.49–2.04)
2	Low SDI	6830.18 (3590.66–11881.16)	10633.7 (6562.69–14060.25)	0.56	2.44 (1.28–4.25)	1.82 (1.12–2.41)	−0.95 (−1.02--0.87)
3	Low-middle SDI	14506.55 (9604.96–22099.07)	18137.88 (12426.3–22433.14)	0.25	2.45 (1.63–3.74)	2.37 (1.63–2.93)	0.08 (−0.02–0.18)
4	Middle SDI	46992.14 (34616.76–62699.16)	93696.82 (57157.35–129568.17)	0.99	6.15 (4.53–8.2)	12.51 (7.63–17.29)	3.3 (2.87–3.73)
5	High-middle SDI	49026.2 (40494.01–59169.43)	101507.01 (59475.24–145311.38)	1.07	13.24 (10.94–15.98)	33.46 (19.61–47.9)	4.39 (3.89–4.89)
6	High SDI	61264.17 (57922.56–64830.04)	60995.49 (54323.09–68218.1)	0	24.38 (23.05–25.8)	26.21 (23.34–29.31)	0.57 (0.32–0.82)
7	High-income Asia Pacific	11061.75 (9340.73–13407.4)	8999.04 (7509.28–10488.43)	−0.19	21.98 (18.56–26.64)	29.23 (24.39–34.07)	1.03 (0.64–1.43)
8	High-income North America	25693.99 (24215.46–27359.78)	19436.2 (17648.12–21442.96)	−0.24	31.44 (29.63–33.48)	21.7 (19.71–23.94)	−0.85 (−1.1–−0.6)
9	Western Europe	35053.02 (32623.36–37744.04)	33643.1 (30567.52–37349.16)	−0.04	35.64 (33.17–38.38)	36.68 (33.33–40.72)	0.07 (−0.28–0.43)
10	Australasia	1075.39 (908.87–1286.44)	1730.82 (1399.42–2103.21)	0.61	17.14 (14.49–20.51)	22.95 (18.56–27.89)	1.53 (0.93–2.13)
11	Andean Latin America	964.83 (745.87–1347.6)	2893.68 (1647.79–4178.63)	2	5.09 (3.93–7.11)	12.22 (6.96–17.65)	3.68 (3.44–3.92)
12	Tropical Latin America	2107.16 (1870.08–2346.23)	3026.46 (2424.14–3728.65)	0.44	3.04 (2.7–3.39)	4.55 (3.64–5.6)	2 (1.74–2.25)
13	Central Latin America	4887.33 (4559.44–5307.73)	8212.24 (6683.21–10456.31)	0.68	5.91 (5.52–6.42)	9.63 (7.84–12.26)	2.24 (1.85–2.64)
14	Southern Latin America	1010.68 (892.33–1158.1)	1826.13 (1474.84–2277.68)	0.81	5.22 (4.6–5.98)	9.36 (7.56–11.67)	2.5 (2.2–2.8)
15	Caribbean	862.04 (676.5–1149.84)	782.05 (609.77–999.07)	−0.09	5.71 (4.48–7.62)	5.12 (4–6.55)	−0.01 (−0.15–0.14)
16	Central Europe	1988.92 (1765.81–2248.77)	2077.14 (1696.95–2562.83)	0.04	5.06 (4.5–5.73)	8.82 (7.2–10.88)	2.35 (2.04–2.67)
17	Eastern Europe	6719.55 (5904.11–7737.73)	4169.16 (3665.47–4,736)	−0.38	9.99 (8.78–11.5)	9.03 (7.94–10.26)	0.83 (−0.04–1.71)
18	Central Asia	1444.71 (1202.84–1728.82)	1532.46 (1197.46–2032.39)	0.06	4.57 (3.81–5.47)	4.43 (3.46–5.87)	0.3 (−0.12–0.72)
19	North Africa and Middle East	8113.76 (5222.7–12152.88)	16350.05 (9509.93–21827.58)	1.02	4.59 (2.95–6.87)	6.91 (4.02–9.23)	2.21 (1.91–2.5)
20	South Asia	10445.52 (6195.55–16645.22)	10543.06 (7152.97–13843.3)	0.01	1.93 (1.14–3.07)	1.54 (1.05–2.03)	−0.74 (−0.84–−0.63)
21	Southeast Asia	7765.36 (5031.67–12113.72)	10498.61 (7069.97–13156.04)	0.35	3.53 (2.29–5.51)	4.58 (3.08–5.74)	1.03 (0.9–1.15)
22	East Asia	53869.63 (37322.74–74646.47)	149267.13 (79096.59–222298.87)	1.77	11.71 (8.11–16.22)	43.27 (22.93–64.44)	6.02 (5.31–6.72)
23	Oceania	36.34 (18.59–65.93)	71.27 (37.66–126.72)	0.96	1.08 (0.55–1.96)	1.12 (0.59–1.98)	0.08 (−0.16–0.31)
24	Central Sub-Saharan Africa	403.49 (196.48–748.35)	692.6 (382.17–1021.74)	0.72	1.3 (0.63–2.41)	0.94 (0.52–1.39)	−0.69 (−0.84–−0.55)
25	Eastern Sub-Saharan Africa	3359.48 (1786.5–5691.07)	5114.9 (3095.41–7566.83)	0.52	3.03 (1.61–5.13)	2.25 (1.36–3.32)	−1 (−1.14–−0.86)
26	Southern Sub-Saharan Africa	338.47 (241.41–484.37)	517.17 (352.94–663.79)	0.53	1.28 (0.91–1.83)	1.65 (1.13–2.12)	1.22 (0.84–1.6)
27	Western Sub-Saharan Africa	1528.68 (754.17–2332.85)	3711.94 (1405.35–5564.78)	1.43	1.42 (0.7–2.17)	1.38 (0.52–2.07)	0.21 (0.09–0.34)
Age	<5 years	106172.66 (88471.54–133096.57)	168879.41 (103720.68–240540.11)	0.59	17.13 (14.27–21.47)	25.66 (15.76–36.55)	2.26 (1.94–2.58)
	5–9 years	33752.4 (28602.52–39943.52)	55206.41 (39272.92–67630.64)	0.64	5.78 (4.9–6.85)	8.04 (5.72–9.84)	1.24 (1.06–1.42)
	10–14 years	21464.47 (18494.27–24492.6)	36905.2 (28585.31–42795.08)	0.72	4.01 (3.45–4.57)	5.54 (4.29–6.42)	1.12 (1.02–1.23)
	15–19 years	17340.59 (14389.9–19743.16)	24104.2 (17276.61–28321.34)	0.39	3.34 (2.77–3.8)	3.86 (2.77–4.54)	0.5 (0.42–0.59)

### SDI regional level

In 2021, children and adolescents in middle-SDI regions had the highest number of ALL cases of prevalence, incidence, deaths, and DALYs, with 93696.82 (95% UI: 57157.35–129568.17), 17798.08 million (95% UI: 11673.78–23203.49), 8032.15 (95% UI: 5632.43–9668.12), and 650588.69 million (95% UI: 454979.73–789360.98), respectively. These cases account for about one-third of the all cases globally. High-middle SDI have the highest prevalence and incidence rates, and high-SDI areas have the lowest rates of death and DALYs. The number of prevalent cases for high-SDI regions globally in 2021 remained unchanged from 1990. In contrast, the prevalence of cases for each of the other SDI regions demonstrated an increase. Specifically, the cases for prevalence, incidence, deaths, and DALYs for low-SDI countries exhibited a notable rise. Conversely, the number of incidences, deaths, and DALYs is negative increases globally for all other SDI regions. The EAPC in incidence for high-SDI regions remained stable between 2019 and 2021 (EAPC: 0). In comparison, the incidence, deaths, and DALYs for each of the other SDI regions showed negative growth. Overall, there is a trend of increasing EAPC for both incidence and prevalence as one moves from low to high-middle SDI regions, while the EAPC for deaths and DALYs exhibits a decreasing trend (Table 1; Figure 1; Supplementary Tables S1, S2).

**Figure 2:**
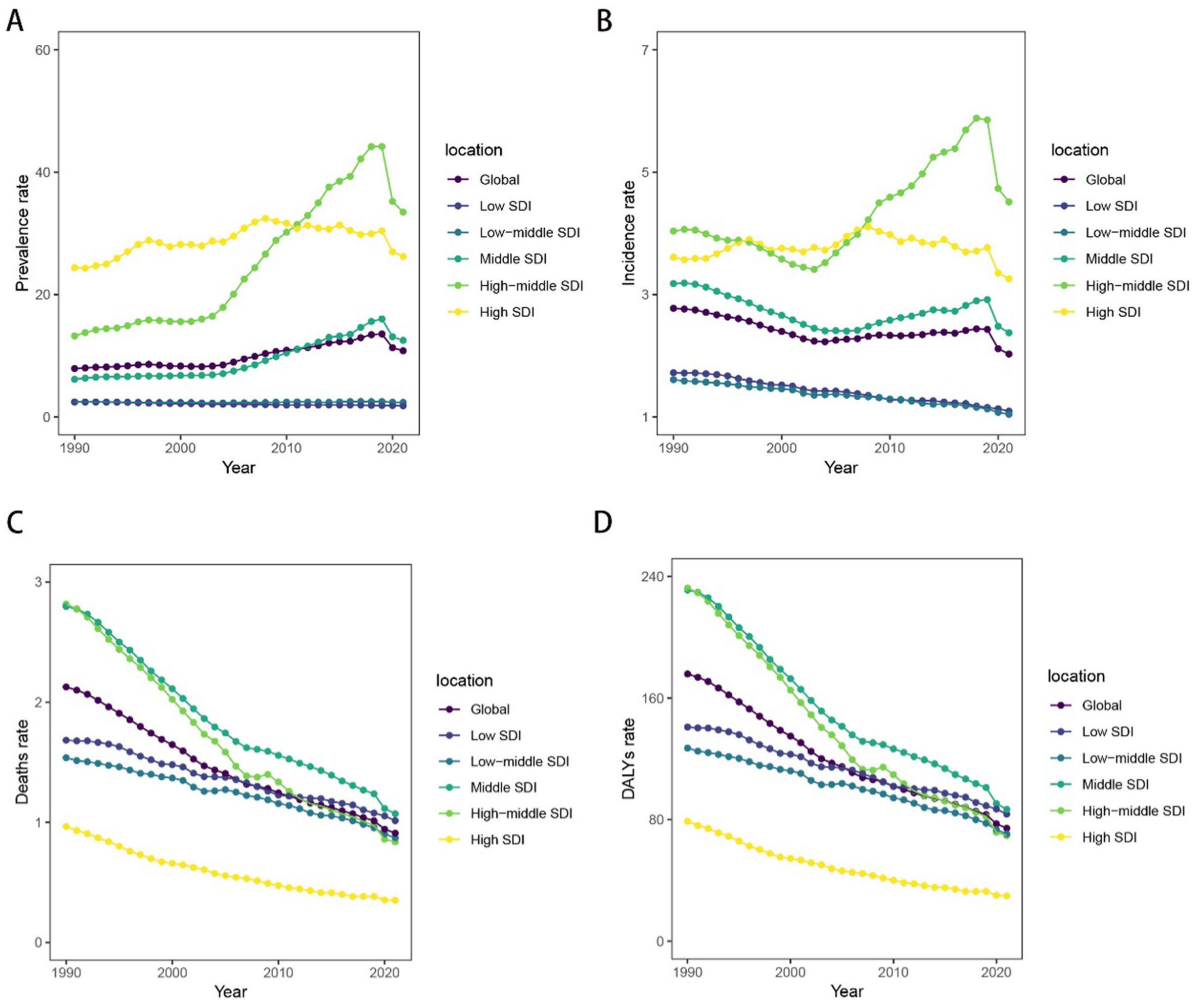
Temporal trend of childhood and adolescent ALL burden in global and 5 territories from 1990 to 2021. **(A)** The rates of prevalence from 1990 to 2021. **(B)** The rates of incidence from 1990 to 2021. **(C)** The rates of death from 1990 to 2021. **(D)** The rates of DALYs from 1990 to 2021.

Trends in mortality and DALYs downward from 1990 to 2021. In contrast, the prevalence and incidence rates for middle and middle-high SDI regions have shown an upward trend since 2005, with medium SDI regions experiencing a more pronounced increase. However, between 2019 and 2021, the prevalence and incidence rates for middle and middle-high SDI regions declined. The prevalence and incidence rates for low and low-middle SDI regions have remained relatively stable and are lower than those of other higher SDI regions. The prevalence and incidence rate of high SDI exhibit slight fluctuations while maintaining a consistently elevated level. However, deaths rate and DALYs are among the lowest (Figure 2).

### GBD regional level

In 2021, prevalence and incidence rates are highest in East Asia, with 43 (95% UI, 22 to 64) cases and 5.98 (95% UI, 3.27 to 8.72) cases per 100,000 population, respectively. Andean Latin America had the highest rates of death and DALYs, with 2 (95% UI, 1.34 to 2.59) deaths and 161 (95% UI, 107 to 210) DALYs. In contrast, the lowest prevalence and incidence rates region is Central Sub-Saharan Africa, with 0.94 (95% UI, 0.52 to 1.39) cases and 0.6 (95% UI, 0.33 to 0.87) cases per 100,000 population. The lowest deaths and DALYs rate are Australasia, with 0.29 (95% UI, 0.25 to 0.25) deaths and 24.91 (95% UI, 21.73 to 28.7) DALYs per 100,000 population. From 1990 to 2021, the percentage change in the number of incidences, deaths, and DALYs decreased across the majority of regions, with the exceptions of Oceania and Central, Eastern, Southern, and Western Sub-Saharan Africa, specifically Western Sub-Saharan Africa, where the percentage changes were 1.04, 0.93, and 0.91%, respectively. Additionally, while EAPC for deaths and DALYs declined overall during this period, Sub-Saharan Africa exhibited an increase, with EAPC values of 0.447 for deaths and 0.45 for DALYs (Table 1).

### Countries level

**Figure 3:**
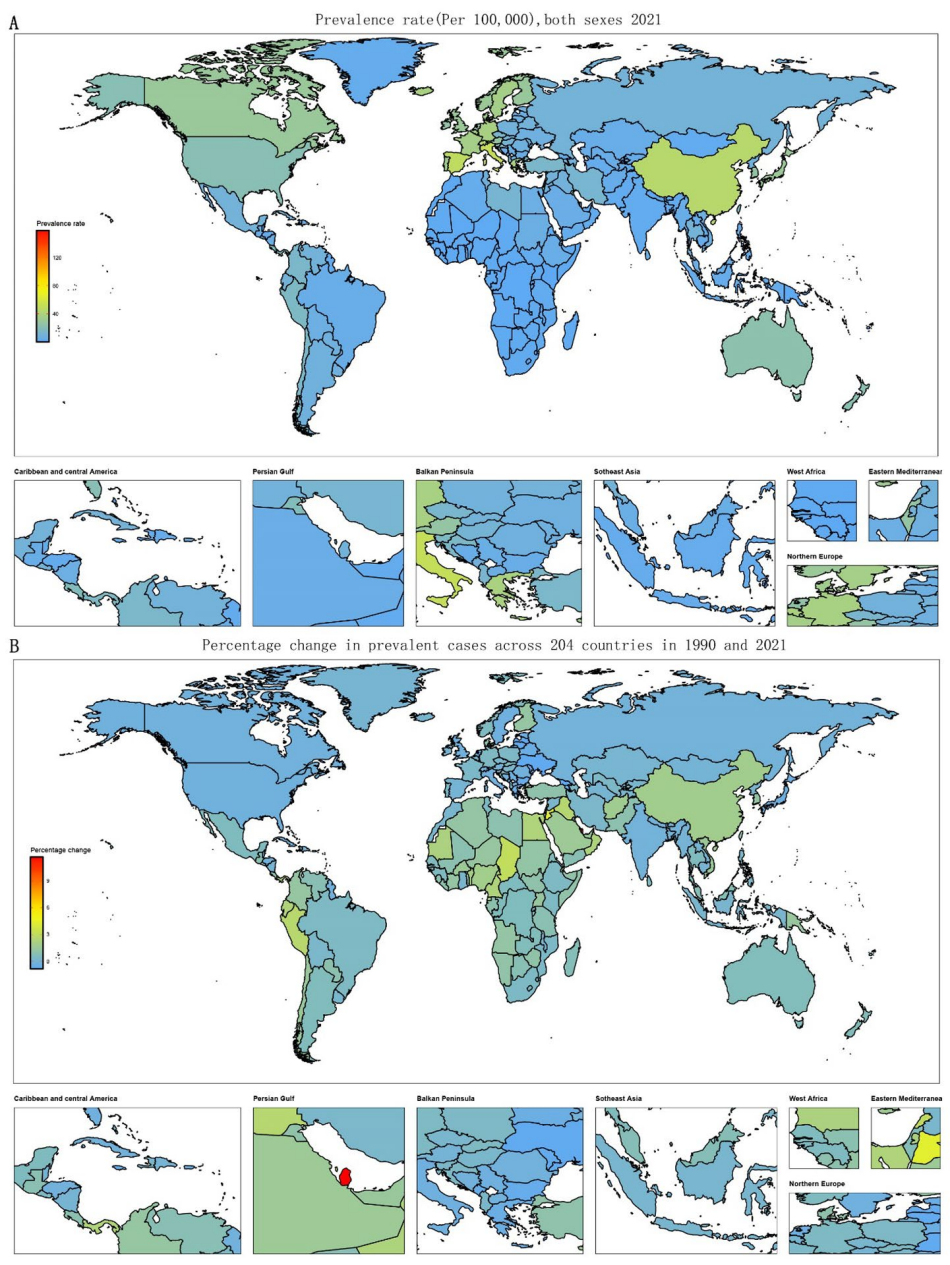
Temporal trend of childhood and adolescent ALL burden globally. **(A)** Prevalence rates across 204 countries from 1990 to 2021. **(B)** Percentage change in prevalent cases across 204 countries in 1990 and 2021.

Between 1990 and 2021, about 72.5% of countries showed a downward trend in deaths and DALYs cases among ALL in children and adolescents. In 2021, Monaco reported the highest prevalence and incidence of ALL in children and adolescents among 204 countries, with 157.9 and 18.9 cases per 100,000 population, respectively. It was followed by Bolivia, San Marino, Italy, Malta, Spain, and China. Haiti reported the highest number of deaths and DALYs among ALL in children and adolescents, with 2.8 deaths per 100,000 and 235.9 DALYs per 100,000, respectively. This was followed by Bolivia. In contrast, Guinea had the lowest deaths of 0.48 cases per 100,000 population. The Cook Islands had the lowest incidence, deaths, and DALYs at 0.20, 0.06 and 5.29 per 100,000 population, respectively. Between 1990 and 2021, Qatar experienced the greatest increase in the percentage change of prevalent and incidence cases, which rose by 11.63 and 4, respectively. Additionally, Chad exhibited the most increases in percentage change in death and DALYs cases, with rises of 2.8 and 2.74. Conversely, Georgia exhibited the most decreases in percentage change in prevalent and incidence cases, with decreases of −0.83 and − 0.87. Moldova exhibited the most decreases in percentage change in death and DALYs cases, with decreases of −0.91 (Figure 3 and Supplementary Figures S1–S3).

### Age and sex patterns, temporal joinpoint analysis

**Figure 4:**
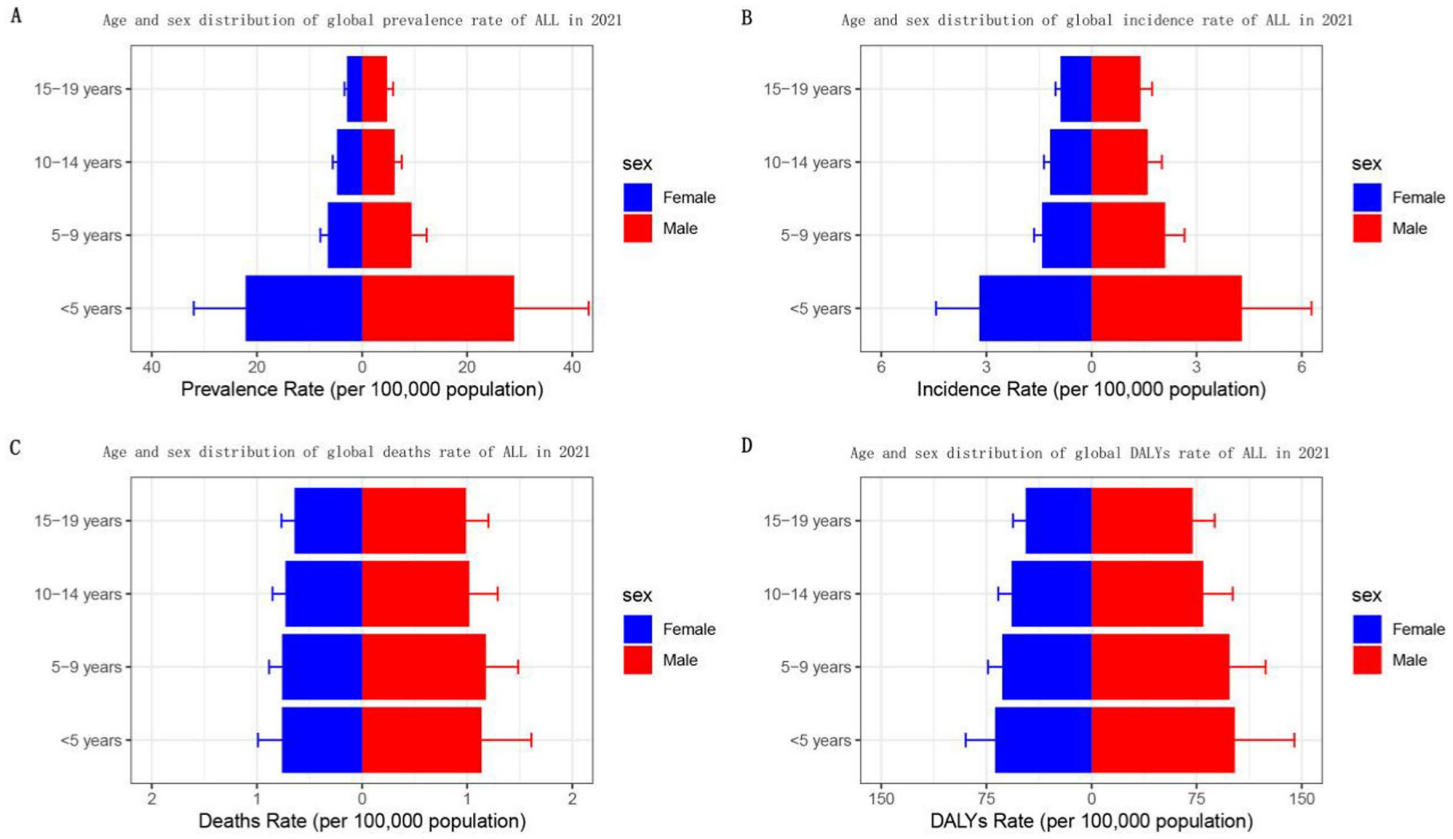
Sex- and age-structured analysis of childhood and adolescent ALL burden in 2021. **(A)** Prevalence rates; **(B)** incidence rates; **(C)** mortality rates; **(D)** DALYs rates.

In 2021, the prevalence and incidence rates of ALL in children and adolescents are significantly higher in males. Specifically, the prevalence ranges from 4.8 to 28.9 per 100,000 population for males and from 2.87 to 22.15 for females. Correspondingly, the incidence rates range from 1.40 to 4.29 per 100,000 population for males, compared to 0.88 to 3.20 for females. Furthermore, both prevalence and incidence rates are notably higher among patients under 5 years of age than in older age groups. As age increases, the prevalence and incidence rates of ALL in children and adolescents tend to decline. Additionally, deaths and DALYs are higher for males than for females. However, the differences in death rates and DALYs by age are not statistically significant. The death rates are recorded at 0.99 to 1.14 per 100,000 population for males and 0.64 to 0.76 per 100,000 population for females, while DALYs range from 72.2 to 102.3 per 100,000 population for males and 47.0 to 68.8 per 100,000 population for females (Table 1; Figure 4; Supplementary Tables S1–S3).

**Figure 5:**
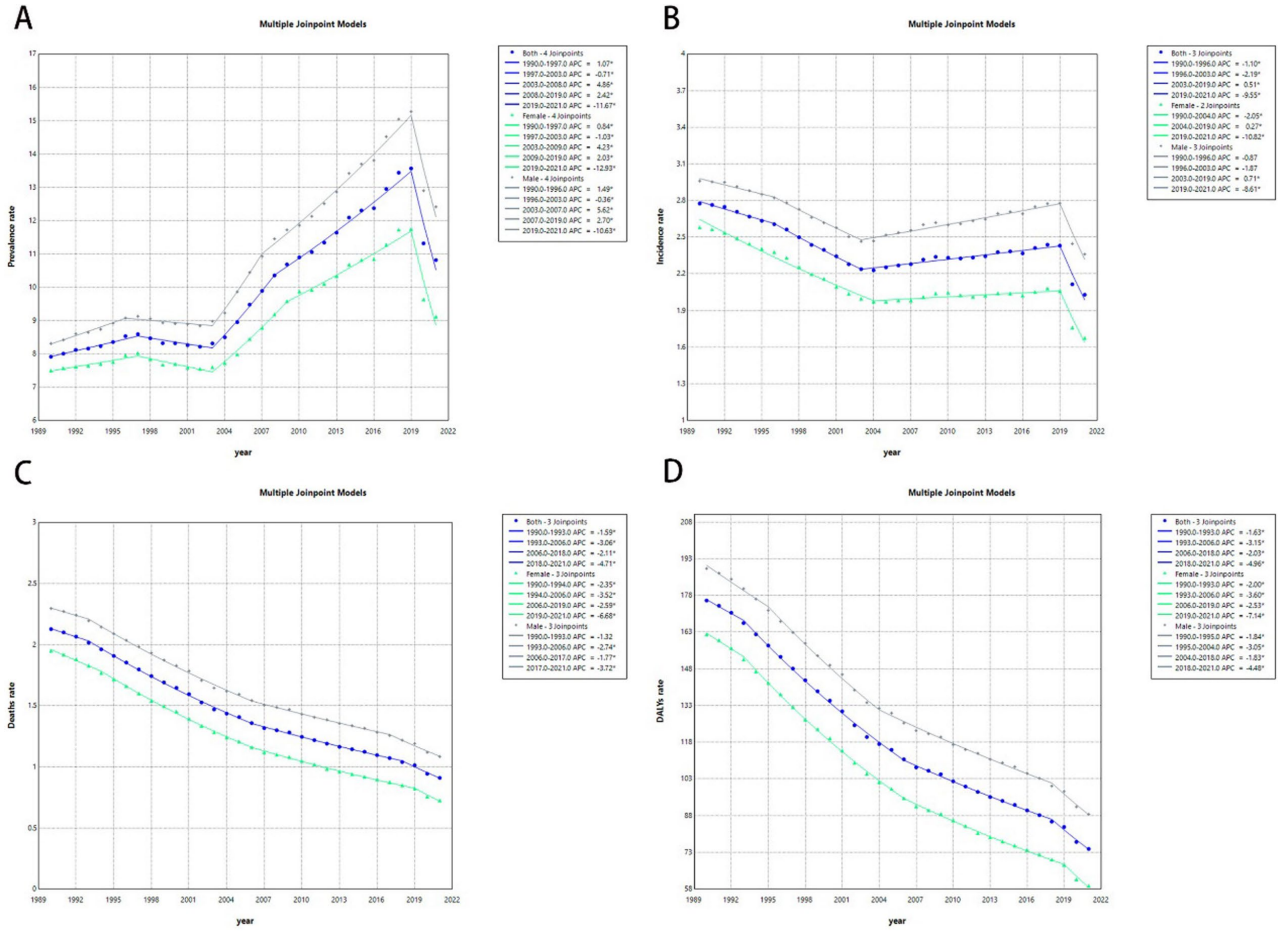
Joinpoint regression analysis of the childhood and adolescent ALL burden temporal trends, 1990–2021. **(A)** Age-standardized prevalence rates. **(B)** Age-standardized incidence rates. **(C)** Age-standardized mortality rates. **(D)** Age-standardized DALYs rates.

From 1990 to 2021, males with ALL consistently exhibited higher prevalence, incidence, mortality, and DALY rates than females across all years. There has been a decline in both death and DALY rates for patients of both sexes. Notably, the prevalence and incidence rates for both sexes began to rise after 2003, with the increase in prevalence being more pronounced than that of incidence. However, between 2019 and 2021, both incidence and prevalence rates of ALL in children and adolescents decreased for both sexes each year (Figure 5).

### The association between burden and SDI

**Figure 6:**
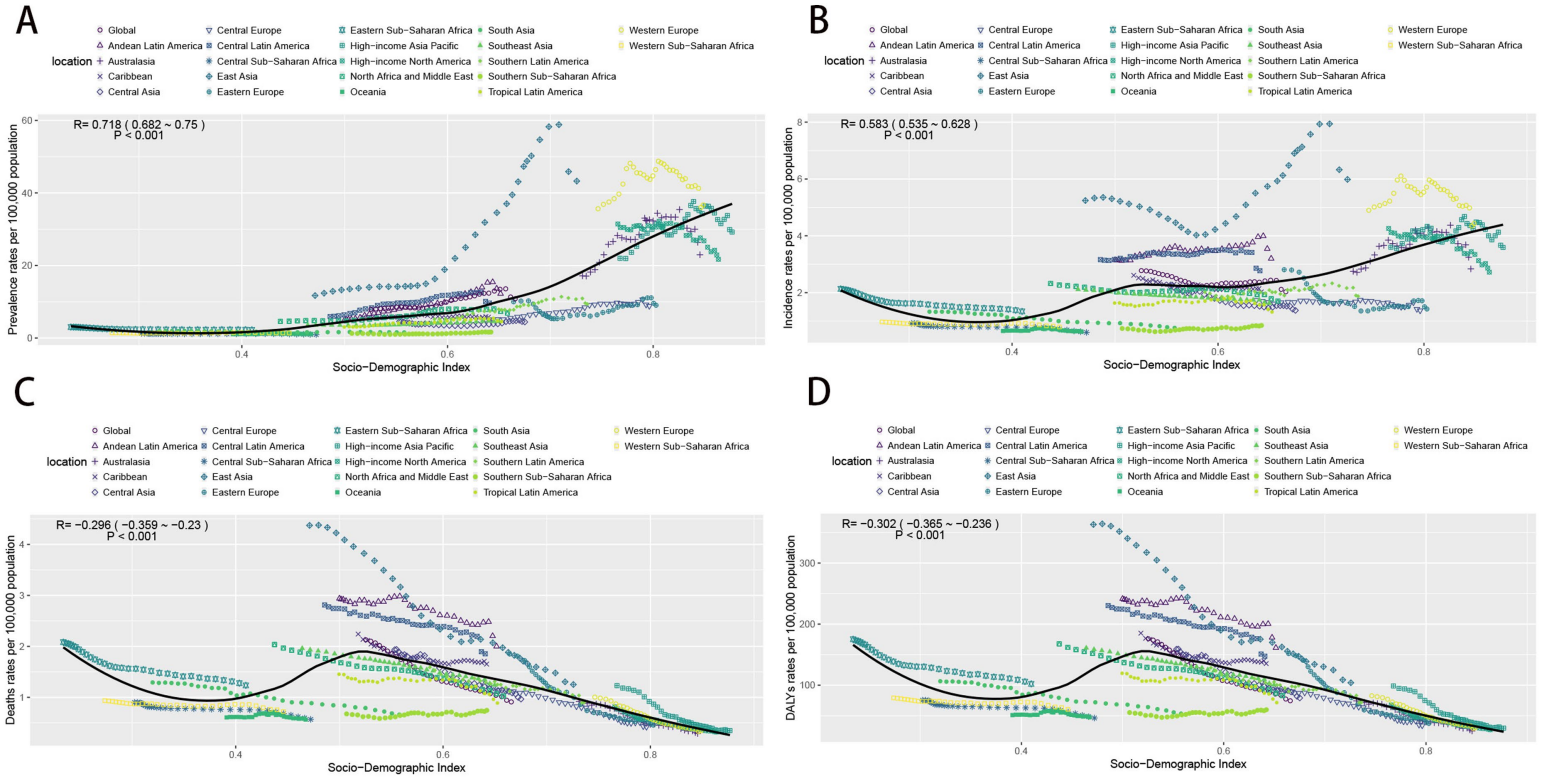
The change trends and correlation analyses of the rates and SDI from 1990 to 2021. **(A)** The change trends and correlation of incidence rate and SDI from 1990 to 2021 in 21 regions. **(B)** The change trends and correlation of incidence rate and SDI from 1990 to 2021 in 21 regions. **(C)** The change trends and correlation of death rate and SDI from 1990 to 2021 in 21 regions. **(D)** The change trends and correlation of DALYs rate and SDI from 1990 to 2021 in 21 regions. SDI, socio-demographic index.

The analysis indicates a positive correlation between prevalence rates and the SDI. Specifically, prevalence rates remained stable and lower when the SDI index was less than 0.5. In contrast, when the SDI index exceeded 0.5, prevalence rates increased in tandem with the index, experiencing a more rapid escalation for SDI indices above 0.7. Notably, East Asia and Western Europe displayed significantly higher prevalence rates than expected, whereas other regions aligned closely with expected values. The incidence, deaths, and DYLAs rates were in the range of the SDI index of less than 0.5, with a decreasing and then increasing trend, bounded by the SDI index of 0.35. For SDI index greater than 0.5, the incidence rates continued to rise slowly as the SDI increased. Conversely, both death and DALY rates for SDI above 0.5 showed a rapid decline. Moreover, East Asia, Western Europe, Andean Latin America, and Central Latin America reported incidence rates that were higher than expected. In contrast, Southern Sub-Saharan Africa, Central Europe, and Eastern Europe recorded incidence and prevalence rates that were lower than anticipated. In terms of deaths and DALYs, East Asia, Andean Latin America, and Central Latin America exhibited rates higher than expected, while Southern Sub-Saharan Africa demonstrated lower than expected (Figure 6).

## Discussion and conclusion

This study presents, for the first time, a comprehensive analysis of global, regional, and national prevalence rates and DALYs associated with ALL in children and adolescents aged 0–19 years from 1990 to 2021. This research systematically accounts for factors such as age, sex, and SDI (socioeconomic development index). We emphasize the necessity of timely enhancing and updating data on the burden of ALL among the global child and adolescent population to equip policymakers with the information required to formulate effective treatment strategies. Over the past three decades, there has been an overall downward trend in the global burden of ALL in children and adolescents, reflecting improvements in treatment. However, as large disparities in burden remain across regions and countries, ongoing monitoring and interventions are needed.

According to our study, the global prevalence of childhood and adolescent ALL in 2021 has increased 60% compared to 1990. The prevalence and incidence rates is decreasing from 2019 to 2021 probably due to COVID-19 (8). However, the incidence, deaths, and DALYs have decreased. Nationally, 72.5% of countries have reported a decline in both death rates and DALYs. The observed reduction in death rates may be attributed to the advancement of modern pediatric treatment protocols, which have significantly enhanced the survival of childhood and adolescent ALL, achieving an estimated overall five-year survival rate of 90% in developed regions (4). Several factors have contributed to these results, including the implementation of extensive collaborative clinical trials and a deeper understanding of the biology of the ALL, and risk-stratified treatment approaches (9). In recent years, new treatment regimens have resulted in higher remission rates for ALL patients with refractory relapses (10). All SDI regions demonstrate a decrease in the number of incidences, deaths, and DALYs, except for the low-SDI regions. Incidence rates show a positive correlation with the index when the SDI exceeds 0.5. Conversely, deaths and DALYs exhibit a negative correlation with the index. Factors contributing to survival in low-SDI regions include limited access to cancer care facilities, delays in the initiation of treatment following symptom onset, and discontinuation in treatment. Many low-income countries lack sufficient resources for laboratory diagnostics and equipment for risk stratification, which hampers or delays accurate diagnosis of ALL in childhood and adolescent (11, 12). Furthermore, inadequate supportive care has been identified as a key factor contributing to decreased overall survival rates among pediatric patients with ALL (5, 13).

From a therapeutic standpoint, unlike other solid tumors in children, ALL is typically treated without surgery or radiotherapy. However, successful management still necessitates experienced medical personnel and appropriate supportive care (2). In 2021, the highest number of prevalence, incidence, deaths, and DALYs occurred in middle-SDI regions. East Asia reported the highest prevalence and incidence cases, whereas South Asia recorded the greatest number of deaths and DALYs. Many challenges persist in effective treatment in childhood and adolescent ALL, including a high frequency of toxicity-related deaths and treatment interruptions (5). Some researchers have noted that age restrictions in pediatric wards or facilities often prevent patients with ALL from accessing specialized care (14, 15). This limitation may negatively impact the treatment of ALL, as pediatric protocols may not be available within adult cancer centers (16). In addition, prevalence and incidence were found to be highest among children under 5 years of age, which represents a distinct demographic compared to the general population (17). Epidemiological and modeling studies have elucidated the dual role of common infections in this context (18). Early-life exposure to microorganisms may confer protective benefits, whereas the absence of such exposure can result in critical secondary mutations triggered by later infections. Childhood ALL can be viewed as a paradoxical consequence of modern societal advancements, where behavioral changes have restricted early microbial exposure, thereby creating an evolutionary mismatch between the immune system’s historical adaptations and contemporary lifestyles (19). Moreover, research indicates that the prevalence of childhood and adolescent ALL is higher in males than in females, potentially attributable to sex-specific tumor suppressor genes (20).

ALL in childhood and adolescent has many clinical manifestations, and the cure is greatly increased by early detection and early medical attention. From the perspective of managing treatment-related toxicity, it is particularly important to reduce treatment intensity in low-risk recurrence populations while simultaneously enhancing supportive care for patients in low-and middle-SDI regions (21, 22). Infections remain a leading cause of mortality during ALL treatment in low-and middle-SDI regions. Therefore, prevention strategies should focus on minimizing excessive myelosuppression and antimicrobial therapies (23).

To reduce deaths and DALYs in low-income countries and regions, we recommend the establishment of national cancer institutes and pediatric tumor registries to enhance patient care and collect demographic data (24). Facilitate diagnosis of disease by reducing the cost of genetic analysis, such as the use of nanopore technology RNA sequencing (25). Selecting the most useful diagnostic methods to stratify patient risk. Reduce the intensity of standard chemotherapy through molecularly targeted therapies and immunotherapy to reduce treatment-related toxicity and improve the prognosis of patients in low-income countries (26). Promote the development of regional frameworks for multidisciplinary collaboration in low-income regions and countries, including training and education of healthcare workers, capacity building, and intensity-escalating treatments (27). Effective access to medicines in low-and middle-income countries can be achieved through collaboration with clinical staff in high-income countries and drug manufacturers (28).

Deaths and DALYs in childhood and adolescent with ALL are expected to decline in the further of immunotherapy and precision medicine era. The next phase will focus not only on optimizing treatment regimens but also on providing sustained attention during and after treatment. This includes targeted psychological support, interventions to promote adherence, reproduction counseling, and long-term survivorship care (29). We anticipate a near future where all children globally have access to timely and accurate diagnoses as well as tolerable and effective treatment regimens designed to maximize cure rates universally.

These findings provide evidence for the future development of age, gender, region, and country-specific intervention strategies in childhood and adolescent ALL. In low-SDI regions and countries, it is essential to consider the influence of socioeconomic factors on ALL outcomes and to address disparities in deaths and DALYs through improved education, healthcare services, and treatment options.

### Limitation

Due to the low level of medical care in some less developed countries, misdiagnosis and underdiagnosis may occur, leading to an underestimation of the burden of disease. In addition, data from global burden of disease (GBD) studies rely heavily on statistical modeling, and many countries lack raw data. It is also important to recognize the lagging nature of GBD data. Therefore, more clinical studies are necessary to validate these findings and provide more accurate and comprehensive assessments.

## Data Availability

The original contributions presented in the study are included in the article/Supplementary material, further inquiries can be directed to the corresponding author.
