# Supplementary material for: Global, regional, and national burden of children and adolescents with acute lymphoblastic leukemia from 1990 to 2021: a systematic analysis for the global burden of disease study 2021
Source: Front Public Health. 2025 Jan 29;13:1525751. doi: 10.3389/fpubh.2025.1525751 (PMC11813771; doi:10.3389/fpubh.2025.1525751)
Supplement: Supplementary file 1 [file Supplementary_file_1.docx]

**Supplement materials**

**
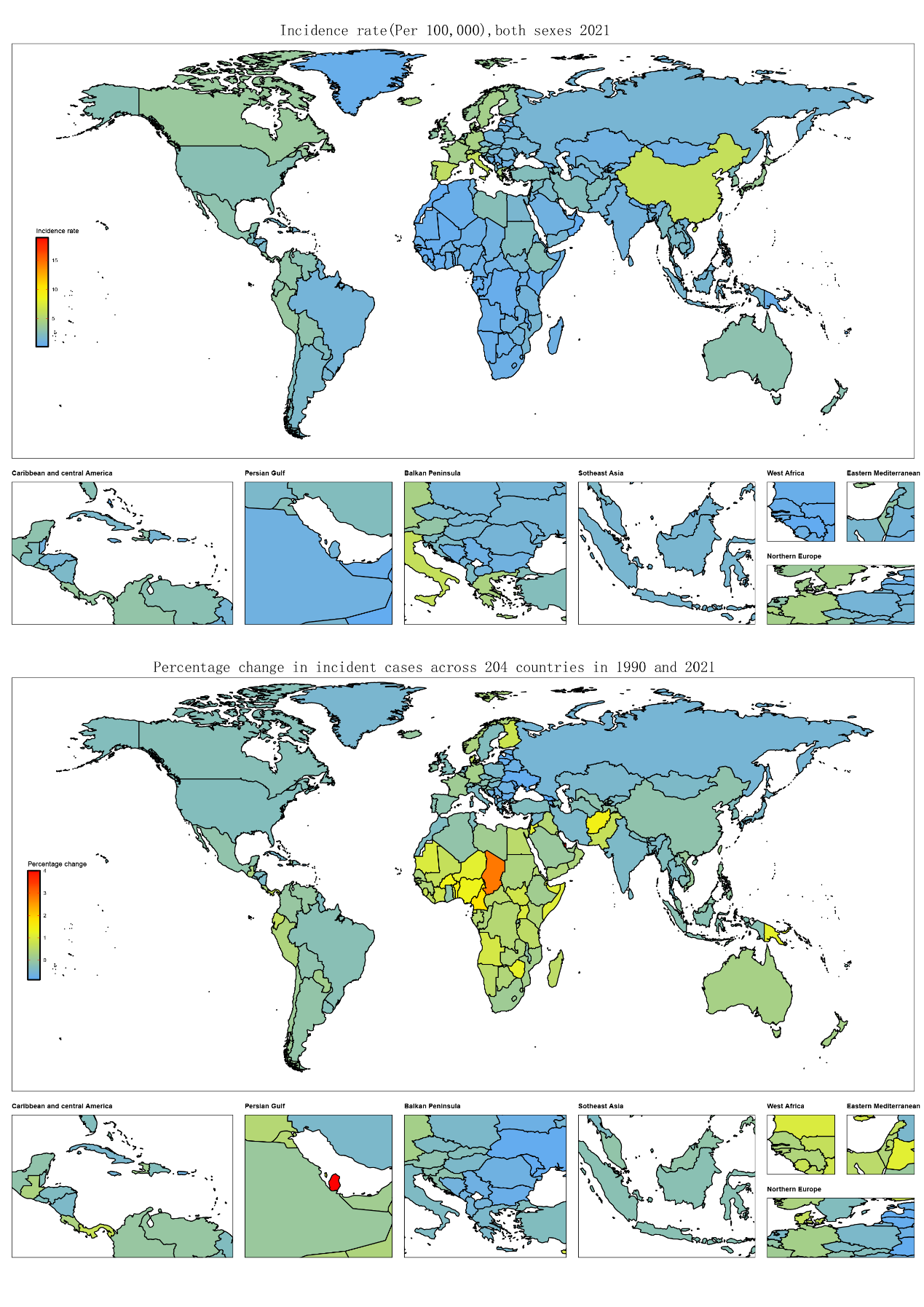
**

Figure S1. Incidence rates and percentage change in incidence cases across 204 countries from 1990 to 2021.

**
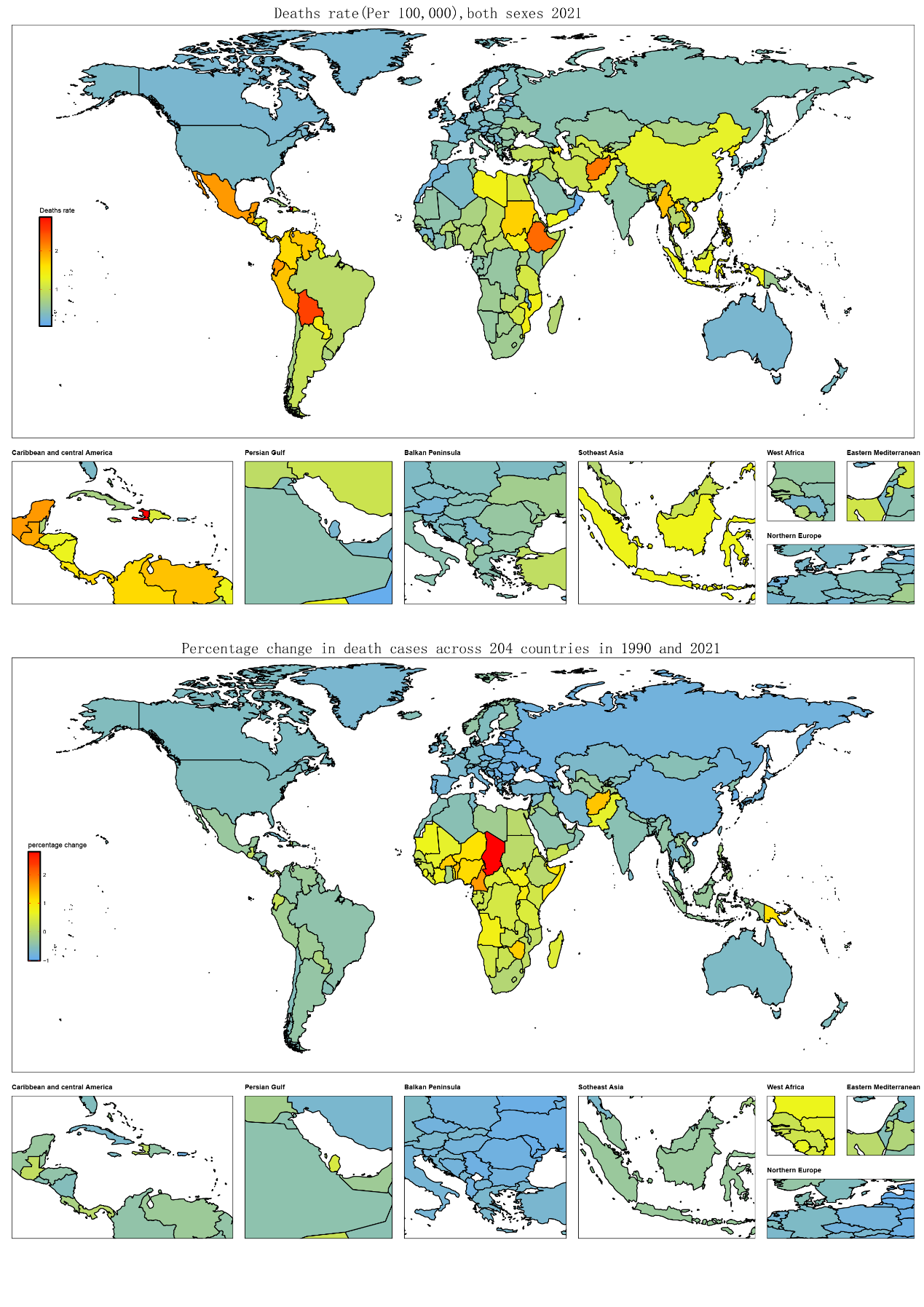
**

Figure S2. Death rates and percentage change in death cases across 204 countries from 1990 to 2021.


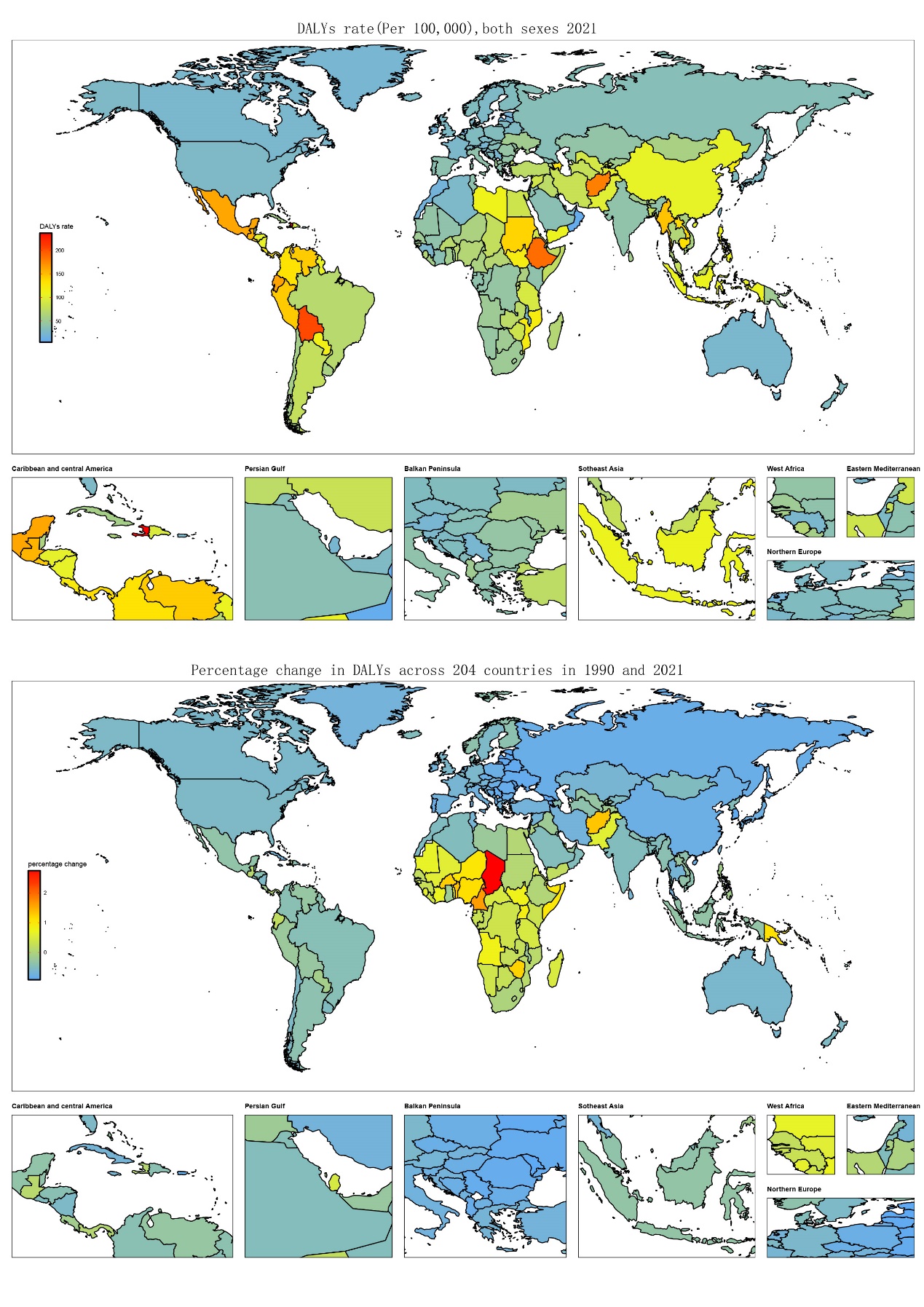
Figure S3. DALYs and percentage change in incidence DALYs across 204 countries from 1990 to 2021.

**Table S1.**The incidence of childhood and adolescent ALL cases and rates in 1990 and 2021, and the trends from 1990 to 2021.

**Table S2.** The deaths of childhood and adolescent ALL cases and rates in 1990 and 2021, and the trends from 1990 to 2021.

**Table S3.** The DALYs of childhood and adolescent ALL cases and rates in 1990 and 2021, and the trends from 1990 to 2021.
